# Overcoming flumatinib resistance in chronic myeloid leukaemia: Insights into cellular mechanisms and ivermectin's therapeutic potential

**DOI:** 10.1111/jcmm.18539

**Published:** 2024-07-24

**Authors:** Jixian Huang, Jie Xiao, Lifeng He, Wenjie Dai, Jian Xiao, Yuquan Li, Ying He, Liang Yu

**Affiliations:** ^1^ Department of Hematology Yuebei People's Hospital Affiliated to Medical College of Shantou University Shaoguan China; ^2^ Physical Examination Center Yuebei People's Hospital Affiliated to Medical College of Shantou University Shaoguan China; ^3^ Medical Research Center Yuebei People's Hospital Affiliated to Medical College of Shantou University Shaoguan China; ^4^ Department of Pharmacology Yuebei People's Hospital Affiliated to Medical College of Shantou University Shaoguan China; ^5^ Department of Hematology Qingyuan People's Hospital The Sixth Affiliated Hospital of Guangzhou Medical University Qingyuan China

## Abstract

Chronic myeloid leukaemia (CML) is a haematological malignancy characterized by the constitutive tyrosine kinase activity of the BCR‐ABL1 fusion protein. Flumatinib, a second‐generation tyrosine kinase inhibitor, has exhibited superior clinical efficacy compared to its precursor, imatinib. However, with increased clinical use, resistance to flumatinib has emerged as a significant challenge. To investigate the mechanisms of flumatinib resistance in CML, we induced the human CML cell line K562 using a flumatinib concentration gradient method in vitro, successfully establishing a flumatinib‐resistant K562/FLM cell line. This cell line exhibited cross‐resistance to imatinib and doxorubicin, but remained sensitive to the antiparasitic agent ivermectin, which possesses antitumoural effects. Through cellular experimentation, we explored the resistance mechanisms, which indicated that K562/FLM cells evade flumatinib cytotoxicity by enhancing autophagy, increasing the expression of membrane transport proteins, particularly P‐glycoprotein, ABCC1 and ABCC4, as well as enhancing phosphorylation of p‐EGFR, p‐ERK and p‐STAT3 proteins. Moreover, it was found that ivermectin effectively suppressed the expression of autophagy and transport proteins in K562/FLM cells, reduced the activity of the aforementioned phosphoproteins, and promoted apoptotic cell death. Collectively, the increased autophagy, higher expression of drug‐efflux proteins and hyperactivation of the EGFR/ERK/STAT3 signalling pathway were identified as pivotal elements promoting resistance to flumatinib. The significant effects of ivermectin might offer a novel therapeutic strategy to overcome flumatinib resistance and optimize the treatment outcomes of CML.

## 1 INTRODUCTION

Chronic myeloid leukaemia (CML) is a malignant neoplasm originating from haematopoietic stem cells in the bone marrow, characterized by clonal proliferation. The aetiology of CML is predominantly attributed to a specific cytogenetic anomaly, known as the Philadelphia chromosome, which arises from the reciprocal translocation of the ABL1 gene on chromosome 9 and the BCR gene on chromosome 22, thus engendering the BCR‐ABL1 fusion oncogene. This aberration culminates in constitutive tyrosine kinase activity, promoting uncontrolled proliferation of leukaemic cells. The advent of tyrosine kinase inhibitors (TKIs) has markedly revolutionized the therapeutic landscape of CML, converting what was once a fatal malignancy into a condition amenable to long‐term management.^1^ Flumatinib, a novel second‐generation TKI developed in China, was approved for marketing in 2019 and is currently listed as a first‐line treatment option for CML. Phase III clinical studies of flumatinib have demonstrated superior therapeutic efficacy compared to imatinib as a first‐line treatment for chronic phase CML, while maintaining a similar safety profile.^2^ Studies indicate that flumatinib is highly effective and well tolerated, not only for first‐time patients^3^, ^4^, ^5^, ^6^, ^7^ but also for those who have not responded well to previous TKI treatments, such as imatinib or dasatinib.^8^, ^9^, ^10^ It can also counteract drug resistance caused by certain ABL1 kinase domain mutations.^11^ Moreover, flumatinib has shown promise in treating Philadelphia chromosome‐positive acute lymphoblastic leukaemia (Ph + ALL),^12^, ^13^, ^14^, ^15^, ^16^, ^17^, ^18^ suggesting its therapeutic benefits may extend beyond CML.

However, as flumatinib is increasingly used in the treatment of leukaemia, some patients have developed resistance to this drug, highlighting the urgent need to establish cell line resistant to flumatinib as in vitro model. This model is essential to understand resistance mechanisms and to find ways to counteract flumatinib resistance. In the light of this, the present study has initiated the establishment of a flumatinib‐resistant cell line, K562/FLM, by exposing the human CML K562 cell line to prolonged low‐dose flumatinib treatment. Furthermore, we investigated the molecular mechanisms underlying flumatinib resistance in K562/FLM cells and identified that ivermectin, an insecticide with unexpected antitumor properties, exhibits significant cytotoxic effects against these resistant cells. This work not only provides experimental insights into the mechanisms of flumatinib resistance in patients with CML but also lays both theoretical and practical groundwork for the development of novel therapeutic strategies for CML treatment.

## 2 MATERIALS AND METHODS

### 2.1 Reagents and antibodies

Flumatinib (purity ≥98%, CAS No. 895519‐90‐1, MW 562.59) was provided by Jiangsu Hansoh Pharmaceutical Grpup Co., Ltd., while imatinib (purity ≥99.9%, CAS No. 152459‐95‐5, MW 493.6), doxorubicin hydrochloride (purity ≥99.6%, CAS No.25316‐40‐9, MW 579.98) and ivermectin (purity ≥98.01%, CAS No. 70288‐86‐7, MW 875.09) were acquired from Med Chem Express. These drugs were dissolved in DMSO and diluted to a concentration of 1 mM, then stored at −80°C. RPMI 1640 medium (C11875500BT), penicillin–streptomycin (15140122) and fetal bovine serum (10091148) were purchased from Gibco. The Cell Counting Kit‐8 (C0038), Annexin V‐FITC Apoptosis Detection Kit (C1062), Cell Cycle and Apoptosis Analysis Kit (C1052), primary antibodies for phospho‐ERK (AF5818), ERK (AF1051), phospho‐EGFR (AF1429), EGFR (AF1330), mTOR (AF1648) and p62/SQSTM1 (AF0279) were purchased from Beyotime Biotechnology. The phospho‐mTOR (67778‐1‐Ig), P glycoprotein (22336‐1‐AP), ABCC1 (67228‐1‐Ig) and ABCC4 (67230‐1‐Ig) antibodies were acquired from Proteintech. The phospho‐STAT3 (PAB36336‐P), STAT3 (MAB51160) and caspase‐3 (PAB30842) antibodies were obtained from Bio‐Swamp, China. GAPDH (2118S) was bought from Cell Signalling Technology.

### 2.2 Establishment of flumatinib‐resistant K562 cell line

K562 cells were cultured in RPMI‐1640 medium supplemented with 10% fetal bovine serum, 100 μg/mL streptomycin, and 100 U/mL penicillin. Incubation occurred at 37°C within a humidified atmosphere containing 5% CO_2_. To induce flumatinib resistance, a gradual selection process was employed whereby cells were exposed to incrementally increased flumatinib concentrations, starting at 0.5 nM. Medium containing the drug was replenished every 2–3 days. Following a stable growth period of 7–14 days, the concentration of flumatinib was escalated by increments of 0.5 nM until the target concentration of 50 nM was achieved, generating the flumatinib‐resistant K562 (K562/FLM) cell line. Thereafter, K562/FLM cells were maintained in RPMI‐1640 medium with 50 nM flumatinib for an additional 30 days. This selection process spanned over 2 years.

### 2.3 Evaluation of cell resistance via the CCK‐8 assay

Cells in logarithmic growth phase were seeded into 96‐well plates at a density of 5 × 10^3^ cells per well, in a volume of 100 μL of culture medium with respective drug concentrations. Following a 72‐h incubation, cell viability was assessed by adding 10 μL of CCK‐8 solution to each well and incubating for 2 more hours. Optical density was measured at 450 nm using a microplate reader. Half‐maximal inhibitory concentration (IC50) values were determined using GraphPad Prism software (version 9.3), and resistance indices (RI) were calculated as follows: RI = IC50 (K562/FLM)/IC50 (K562).

### 2.4 Detection of BCR‐ABL1 fusion gene, expression levels and kinase domain mutations in cell samples

Cells for FISH analysis were collected, washed twice with PBS and fixed with 0.075 mol/L potassium chloride. Subsequently, cells underwent three cycles of fixation with a methanol: acetic acid solution (3:1) before slide preparation. A total of 1000 interphase cells were counted, and the hybridization signals were recorded. Quantitative real‐time polymerase chain reaction (qPCR) was utilized to measure the expression level of the BCR‐ABL1 fusion transcript in the cell samples. Additionally, a nested PCR approach combined with Sanger sequencing was used to screen for ABL1 kinase mutations in K562/FLM cells.

### 2.5 Western blot analysis to assess protein expression

Total protein was extracted from the cells using RIPA lysis buffer on ice. The protein concentration was determined using the BCA method. Equal amounts of protein were loaded onto a 10% SDS‐PAGE gel and separated by electrophoresis. The proteins were then transferred to a membrane, blocked and incubated overnight at 4°C with the primary antibody. After incubation with the secondary antibody at room temperature for 1 h, membranes were treated with enhanced chemiluminescence substrate and imaged using a chemiluminescence detection system.

### 2.6 Immunofluorescence detection of LC3B expression

Cells from different treatment groups were fixed with 4% paraformaldehyde according to the instructions of the fluorescence reagent. The cells were washed with TBS containing 0.1% Triton‐X‐100 (TBSTx), blocked with TBSTx containing 5% BSA and incubated overnight at 4°C with the primary antibody. After a 1‐h room temperature incubation with secondary antibody, cells were washed, stained with DAPI and mounted on slides for visualization and photography under an inverted fluorescence microscope.

### 2.7 Annexin‐V/PI double staining and flow cytometry for apoptosis detection

Following harvest, cells were washed with PBS and resuspended in annexin‐binding buffer. Annexin‐V and propidium iodide (PI) dyes were added sequentially, followed by incubation in the dark for 15 min. Cell apoptosis was detected using flow cytometry.

### 2.8 Cell cycle analysis by flow cytometry

After washing with ice‐cold PBS, cells were fixed with 75% ethanol at 4°C for more than 4 h. The cells were then washed twice with cold PBS, stained with PI solution, and incubated in a dark, temperature‐controlled bath at 37°C for 30 min. Samples were stored on ice and protected from light until analysed by flow cytometry on the same day.

### 2.9 Statistical analysis

The images obtained from western blot analysis were processed using the ImageJ software. Statistical analysis of the experimental data was performed using GraphPad Prism 9.3. Normality and homogeneity of variance tests were conducted on all the experimental data, followed by the application of independent sample *t*‐test based on the specific experimental design. The statistical outcomes were presented as mean ± standard deviation (mean ± SD), where *α* = 0.05 was considered the significance level, and *p* < 0.05 indicated statistical significance of the observed differences.

## 3 RESULTS

### 3.1 Resistance of K562/FLM cells to flumatinib and cross‐resistance

**FIGURE 1:**
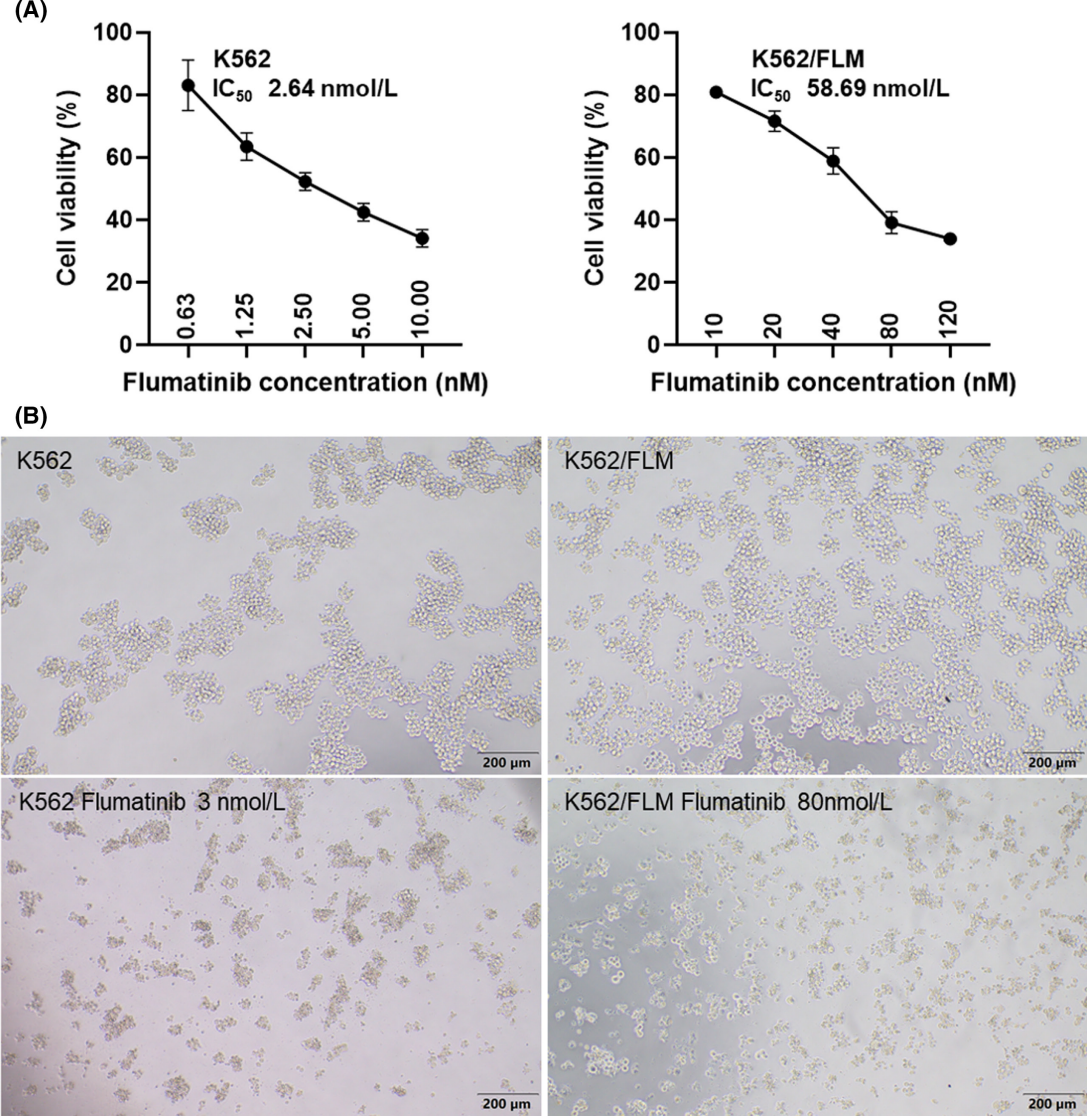
Resistance of K562/FLM cells to flumatinib. (A) 72‐h cytotoxicity of flumatinib against K562 and K562/FLM cells. (B) Morphological observation of K562 and K562/FLM cells after treatment with flumatinib for 72 h.

Differential susceptibility to flumatinib was observed between parental K562 cells and resistant K562/FLM cells (Figure 1). For parental K562 and K562/FLM cells, the 72‐h IC_50_ values of flumatinib were 2.64 and 58.69 nM, respectively, indicating a resistance factor of 22.2. Cross‐resistance was assessed by treating both cell types with varying concentrations of imatinib, doxorubicin and ivermectin for 72 h and determining IC_50_ values. The findings indicate that the K562/FLM cell line exhibits pronounced cross‐resistance to both imatinib and doxorubicin. Specifically, the resistance coefficient to imatinib is >5, denoting a substantial level of resistance. In contrast, the resistance coefficient to doxorubicin is merely 1.8, indicating a relatively lower degree of resistance. Intriguingly, no cross‐resistance was observed for ivermectin in this cell line (Table 1).

**TABLE 1:** Drug sensitivity of K562 cells and K562/FLM cells.

Drug	K562	K562/FLM	Resistance index
Flumatinib	2.643 ± 0.73 nmol/L	58.69 ± 7.09 nmol/L	22.2
Imatinib	0.1067 ± 0.0097 μmol/L	0.5346 ± 0.027 μmol/L	5.01
Doxorubicin	1.004 ± 0.057 μmol/L	1.832 ± 0.34 μmol/L	1.82
Ivermectin	12.72 ± 0.011 μmol/L	13.09 ± 0.01 μmol/L	1.03

### 3.2 Detection of BCR‐ABL1 fusion gene

**FIGURE 2:**
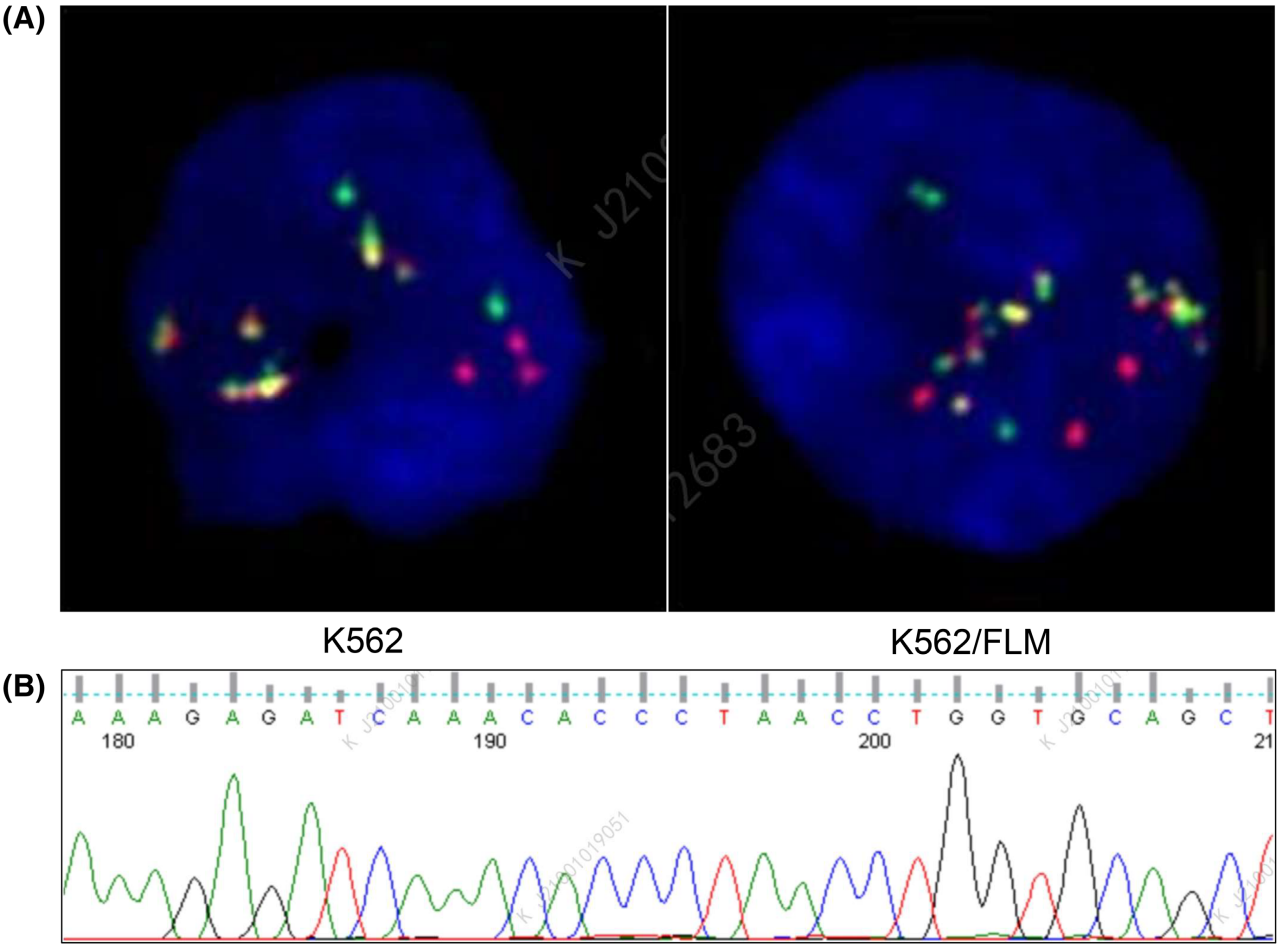
The BCR‐ABL1 fusion gene in K562/FLM cells shows no significant difference compared to K562 cells. (A) FISH detection image showing fusion signals with BCR/ABL translocation probes at the 22q11 and 9q34 loci. (B) Sequencing chromatograms of ABL1 kinase mutations in K562/FLM cells.

The signal characteristics of interphase cells carrying the BCR‐ABL1 fusion gene were observed as follows: 1 green signal (BCR allele), 2 yellow signals (BCR‐ABL1 and ABL1‐BCR fusion genes) and 1 red signal (ABL1 allele). The fluorescence intensity and gene copy number of the BCR‐ABL1 fusion gene in K562/FLM cells showed no significant difference compared to K562 cells (Figure 2A and Table 2). The sequencing results revealed no mutations in the ABL1 kinase domain of the BCR‐ABL1 fusion gene in K562/FLM cells (Figure 2B and Table 3).

**TABLE 2:** Quantitative detection results of BCR‐ABL1 P210 fusion gene in K562 and K562/FLM cells.

Test items	K562	K562/FLM
BCR/ABL1 P210 fusion gene	Positive	Positive
BCR/ABL1 (copy number)	3566256	1177770
ABL1 (copy number)	2454553	824430
BCR/ABL1/ABL1	145.29%	142.86%
IS BCR/ABL1/ABL1	107.52%	105.72%

**TABLE 3:** ABL1 kinase mutation detection results in K562/FLM cells.

Mutation region	T315I	P‐loop	A‐loop	Other mutations
Results	Not detected	Not detected	Not detected	Not detected

### 3.3 Different expression of P‐glycoprotein and autophagy‐related proteins in K562 and K562/FLM cells

**FIGURE 3:**
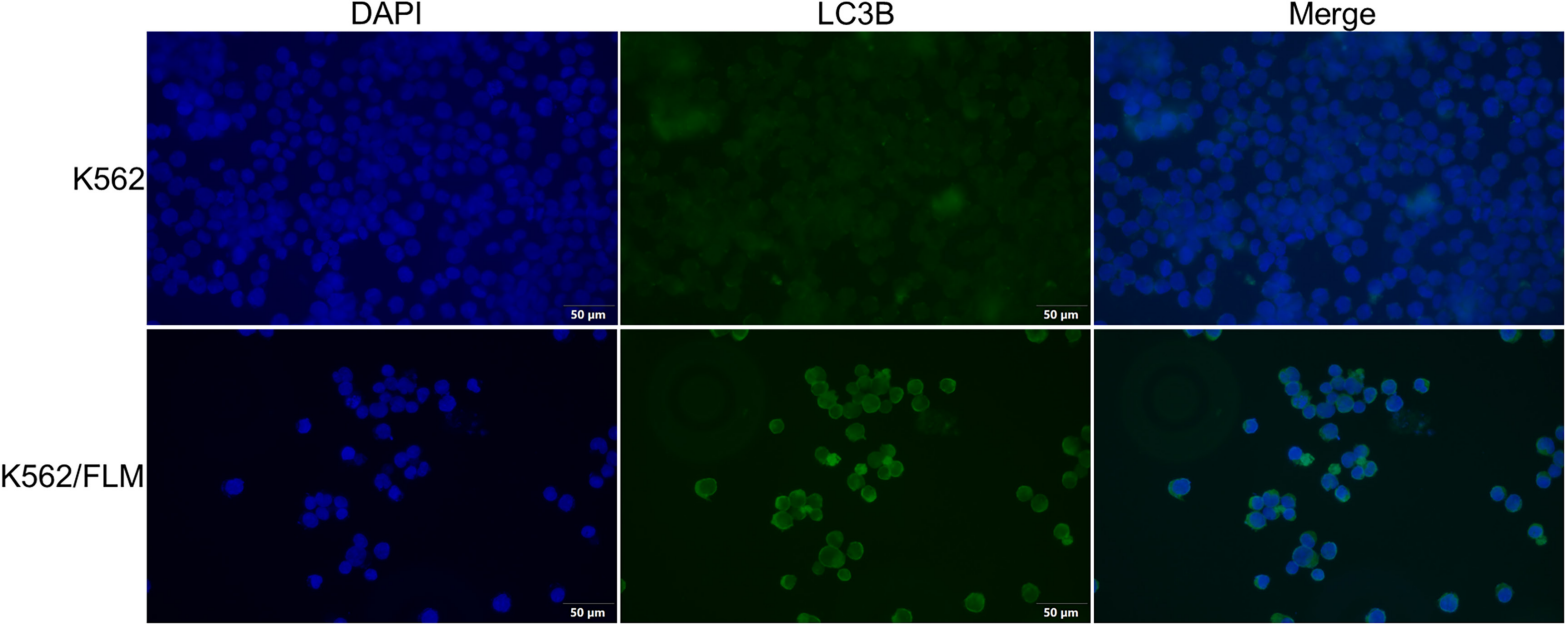
The expression of LC3B is significantly higher in K562/FLM cells compared to K562 cells. Immunofluorescence analysis of K562 and K562/FLM cells using LC3B antibody (green fluorescence). Blue: DAPI for nuclear staining. K562 cells are cultured in RPMI‐1640 medium, while K562/FLM cells are cultured in RPMI‐1640 medium containing 50 nM flumatinib.

To investigate the involvement of autophagic processes in the context of drug resistance, autophagy marker protein LC3B was visualized using fluorescence microscopy. The fluorescence intensity of LC3B was markedly elevated in K562/FLM cells relative to K562 cells, indicative of increased autophagic activity (Figure 3).

**FIGURE 4:**
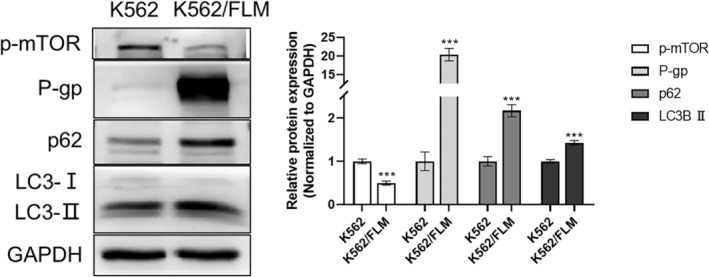
The expression of P‐gp and autophagy‐related proteins is significantly higher in K562/FLM cells than in K562 cells. Immunoblotting of P‐gp, p‐MOTR, p62, LC3 in K562 and K562/FLM cells. GAPDH was used as the loading control. K562/FLM cells are cultured in RPMI‐1640 medium containing 50 nM flumatinib, while K562 cells are cultured in medium without flumatinib. ***p < 0.001 versus the K562 group, Error bars indicate SD (*n* = 3).

This observation was further substantiated by western blotting analysis, which demonstrated a significant upregulation of autophagy‐related proteins LC3‐II and p62 in K562/FLM cells. Furthermore, phosphorylated mTOR levels were notably decreased, suggesting a higher level of autophagy in K562/FLM cells (Figure 4).

P‐glycoprotein (P‐gp) is known to function as a multidrug efflux pump, actively expelling a variety of chemotherapeutic agents from cells and thereby contributing to the development of multidrug resistance (MDR). Expression analysis indicated negligible P‐gp expression in K562 cells, whereas K562/FLM cells exhibited pronounced P‐gp overexpression (Figure 4).

### 3.4 Modulation of signalling pathways and drug resistance proteins by flumatinib and ivermectin in K562 and K562/FLM cells

**FIGURE 5:**
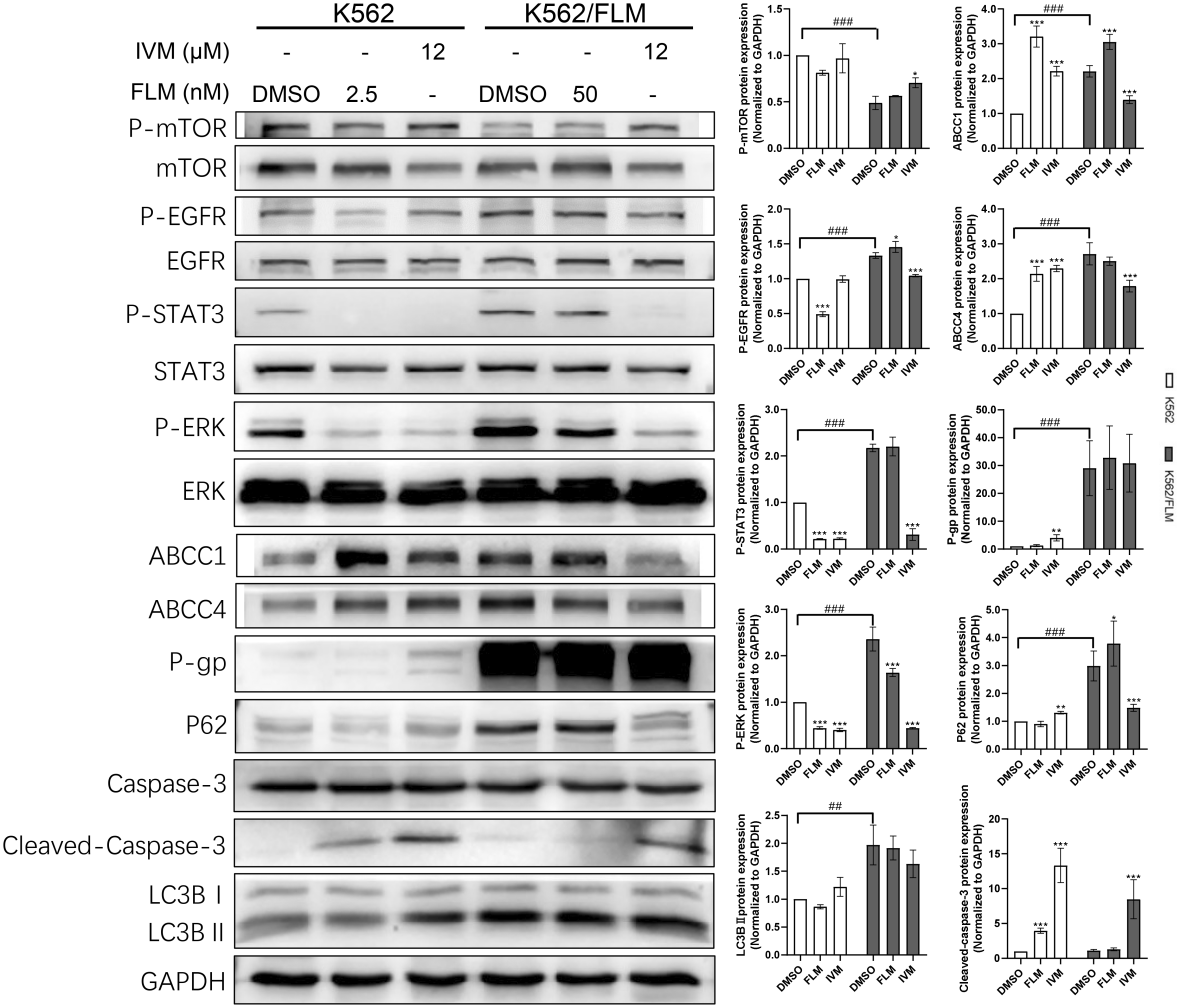
K562/FLM cells resist flumatinib by increasing autophagy levels, high expression of drug‐efflux proteins, and hyperactivation of the EGFR/STAT3/ERK signalling pathway, whereas ivermectin is able to reverse the flumatinib resistance in K562/FLM by inhibiting these mechanisms. The phosphorylation levels of MTOR/EGFR/STAT3/ERK, the protein levels of P‐gp, ABCC1, ABCC4, p62, LC3B in K562 and K562/FLM cells under different administration concentrations of flumatinib and ivermectin. GAPDH was used as the loading control. *p < 0.05, **p < 0.01, ***p < 0.001 versus the DMSO group. ^##^ *p* < 0.01, ^###^ *p* < 0.001 versus the K562 group, Error bars indicate SD (*n* = 3).

Our study findings indicate that relative to K562 cells, K562/FLM cells exhibited a significant upregulation in p‐EGFR, p‐STAT3 and p‐ERK proteins (Figure 5). Additionally, there was a pronounced increase in the expression of the drug resistance‐associated proteins ABCC1 and ABCC4. Treatment with different concentrations of flumatinib for 24 h resulted in a marked reduction in the expression levels of p‐EGFR, p‐STAT3 and p‐ERK proteins in K562 cells, accompanied by the cleavage of caspase‐3 and an increase in cleaved caspase‐3, suggesting that flumatinib exerts a significant apoptotic effect on K562 cells. However, in K562/FLM cells, only p‐ERK protein levels were downregulated, indicating a comparatively limited impact of flumatinib, with no significant effect on cellular apoptosis.

Furthermore, we evaluated the effects of flumatinib on autophagy and drug resistance‐related proteins in both cell types. In K562 cells, the expression of p‐mTOR was inhibited, and the levels of ABCC1 and ABCC4 proteins significantly escalated, without substantial changes observed in other autophagic or drug resistance‐related proteins. In contrast, K562/FLM cells did not demonstrate noticeable changes in autophagic or drug resistance‐related proteins, suggesting an adaptation to high concentrations of flumatinib.

Upon administering an equivalent concentration of ivermectin to both cell types, the expression levels of the resistance proteins ABCC1, ABCC4 and P‐gp in K562 cells were considerably elevated, and an increase in apoptosis was indicated by the upregulation of LC3B and the pronounced inhibition of p‐STAT3, p‐ERK, along with a notable increase in cleaved caspase‐3. Conversely, in K562/FLM cells, a substantial reduction occurred in the phosphorylation levels of p‐EGFR, p‐STAT3 and p‐ERK (Figure 5). Although P‐gp levels did not change markedly, the expression of ABCC1 and ABCC4 was substantially suppressed; p‐MTOR expression was elevated, and p62 and LC3B levels were significantly decreased along with an increase in cleaved caspase‐3, suggesting apoptosis. Collectively, these results demonstrate that ivermectin induces apoptosis in both cell types, albeit through distinct mechanisms of action.

### 3.5 Changes in cell cycle and apoptosis levels in K562 and K562/FLM cells after flumatinib and ivermectin treatment

**FIGURE 6:**
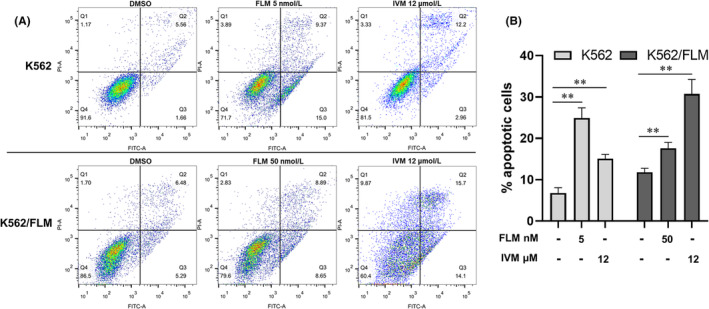
Comparative effects of flumatinib and ivermectin on apoptosis rates in K562 and K562/FLM cells after 24‐h treatment. (A) Flow cytometry was performed after Annexin V‐FITC/PI staining. (B) Results showed the percentage of apoptotic cells. *p < 0.05, **p < 0.01 compared with control, Error bars indicate SD (*n* = 3).

After 24‐h treatment with 5 nM flumatinib, K562 cells demonstrated a remarkable increase in apoptosis rate, whereas K562/FLM cells showed only a modest 6% increase in apoptosis when treated with 50 nM flumatinib, highlighting their tolerance to flumatinib. Following treatment with 12 μM ivermectin for 24 h, apoptosis in K562 cells increased by 8%, while the mortality rate exhibited a 2.16% rise. In contrast, K562/FLM cells demonstrated a more pronounced increase in apoptosis at 18.03% and an 8% augmentation in mortality rate. These findings suggest that a 24‐h exposure to 12 μM ivermectin inflicts greater cytotoxic damage on K562/FLM cells compared to K562 cells. (Figure 6).

To investigate the effects of flumatinib and ivermectin on cell cycle progression, flow cytometric analysis was conducted on K562 and K562/FLM cells after drug treatment. In untreated control K562 cells, the distribution of cell cycle phases was 42.06% in G1, 49.35% in S and 8.59% in G2 phase. Following treatment with 5 nM flumatinib for 24 h, we observed a substantial increase in the proportion of cells in G1 phase to 71.76%, a decrease in the S phase to 28.24% and no detectable cells in the G2 phase, suggesting that the drug induces G1 phase cell cycle arrest. In contrast, treatment with 12 μM ivermectin resulted in a slight increase in G1 phase to 48.01% and a substantial increase in cells in G2 phase to 49.20%, with a drastic decrease in S phase to 2.79%, implying that the drug may induce G2 phase cell cycle arrest.

**FIGURE 7:**
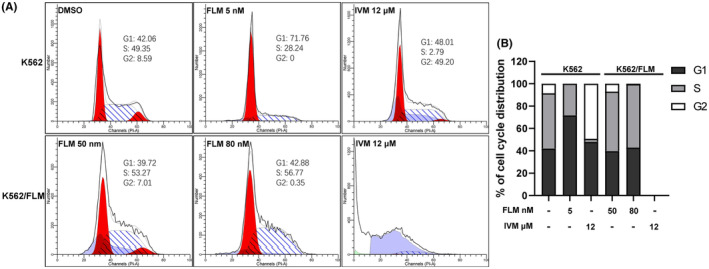
Differential effects of flumatinib and ivermectin on cell cycle progression in K562 and K562/FLM cells. (A) Cell‐cycle distribution was measured by flow cytometry using PI. (B) The percentage of cells in G1, G2 and S phase in K562 and K562/FLM Cells without or with the treatment of flumatinib and ivermectin for 24 h.

In K562/FLM cells, using a culture medium with 50 nM flumatinib as a control, the cell cycle distribution showed 39.72% in G1, 53.27% in S and 7.01% in G2 phase. Even under 80 nM flumatinib treatment, the distribution of cell cycle phases in this resistant cell line was similar to the control group, with 42.88% in G1, 56.77% in S and decreased G2 phase to 0.35%, further indicating its resistance to flumatinib. However, unlike the K562 cells, K562/FLM cells treated with 12 μM ivermectin exhibited extensive cell damage, with 85.17% of cells presenting as Debris, indicating widespread cell death induced by ivermectin at this concentration (Figure 7).

## 4 DISCUSSION

Tumour resistance is commonly categorized into intrinsic resistance, where patients exhibit non‐responsiveness to anticancer drugs at the outset of treatment, and acquired resistance, which arises over time through a reduction in drug sensitivity, ultimately leading to tumour recurrence and metastasis. Traditional concepts suggest that drug resistance arises from three primary mechanisms^19^: (i) issues with transporter proteins involved in drug uptake, reducing the cellular intake of drugs; (ii) intracellular alterations following drug exposure, diminishing the cytotoxicity of the drug; and (iii) enhanced cellular metabolism and efflux capacity for the drug.

In this study, the K562/FLM cell line, established through gradual induction in the presence of flumatinib, exhibited stable growth in media containing 50 nM flumatinib, with a notable resistance factor of 22. Additionally, we assessed the sensitivity of these cells to imatinib, doxorubicin and ivermectin. Our findings suggest a substantial increase in resistance to imatinib, a TKI like flumatinib, with a fivefold resistance factor. This parallel resistance profile could be indicative of overlapping mechanisms involved in the acquired resistance to TKIs in K562/FLM cells, potentially involving either the alteration of the drug target, efflux transporter upregulation or signalling pathway modulation.^20^

Interestingly, while resistance to doxorubicin, an anthracycline antibiotic commonly used in chemotherapy, was observed, it was to a lesser extent. This modest increase in resistance could imply a partial overlap of resistance mechanisms between flumatinib and doxorubicin, possibly via shared efflux pathways or adaptive cellular stress responses. The minimal change in susceptibility to ivermectin, an antiparasitic agent with reported antineoplastic properties,^21^, ^22^ indicates a highly specific resistance mechanism to flumatinib with little cross‐resistance to drugs with dissimilar modes of action.

The resistance mechanisms in CML can be classified into BCR‐ABL1‐dependent and independent types.^23^ In this study, BCR‐ABL1 fusion gene expression did not differ between K562/FLM and K562 cells. This may indicate that the development of drug resistance is not directly related to the inhibition of BCR‐ABL1 kinase activity; suggesting that K562/FLM cells could be BCR‐ABL1‐independent resistance. Studies have indicated that autophagy,^24^ drug transporters and alternative signalling pathways^20^ are among the mechanisms responsible for TKI resistance in CML patients.

The overexpression of P‐gp (ABCB1), ABCC1 and ABCC4 in K562/FLM cells suggests the classic MDR phenotype observed in many resistant cancer cells.^25^ P‐gp,^26^ ABCC1 and ABCC4^27^ acts as an ATP‐dependent efflux pump, reducing the intracellular concentration of various chemotherapeutics, including flumatinib, and thus lowering their cytotoxic efficacy.^28^ Despite the sensitivity to ivermectin being unchanged in both cell lines, the implications of P‐gp overexpression on flumatinib resistance are noteworthy and may necessitate combinational approaches targeting P‐gp to sensitize cells to flumatinib.

Furthermore, the increased autophagic flux, as evidenced by the elevated levels of LC3‐II and p62 in K562/FLM cells, in conjunction with reduced mTOR phosphorylation, is indicative of enhanced autophagy. Autophagy has been implicated in promoting survival in cancer cells under stressful conditions, including chemotherapy.^29^, ^30^ Therefore, autophagy may serve as a protective mechanism for K562/FLM cells, allowing them to evade flumatinib‐induced cytotoxicity. Autophagy inhibition could thus be considered as a co‐treatment to sensitize K562/FLM cells to flumatinib.

The upregulation of p‐EGFR, p‐STAT3 and p‐ERK in K562/FLM cells points to altered signal transduction pathways contributing to drug resistance.^20^, ^31^ In particular, the dysregulation of these proteins is implicated in cell survival and proliferation, and their aberrant activation can facilitate resistance mechanisms.^32^, ^33^ It is notable that K562/FLM cells retain the phosphorylation of p‐EGFR and p‐STAT3 despite flumatinib treatment, suggesting that alternative signalling pathways may be compensating for the inhibitory effects of flumatinib on p‐ERK. This necessitates further investigation into the signalling networks in flumatinib‐resistant cells and their potential as therapeutic targets.

Interestingly, it appears that K562/FLM cells are more susceptible to ivermectin‐induced apoptosis and cell cycle arrest than their parental K562 counterparts. Ivermectin's ability to reduce the phosphorylation of EGFR, STAT3 and ERK pathways in K562/FLM cells, in addition to its suppression of drug resistance‐associated proteins ABCC1 and ABCC4 and the induction of cleaved Caspase‐3, highlight its apoptotic effect that overcomes resistance mechanisms.^34^ Furthermore, the elevated p‐MTOR expression, along with the decreased p62 and LC3B levels upon ivermectin treatment, suggests an interplay between autophagy and apoptosis in these cells.^21^

The stark contrast in the ability of flumatinib to induce cell cycle arrest in K562 cells but not in K562/FLM cells reinforces the resistance of the latter to the drug. However, the profound G2/M cell cycle arrest and induction of cell death in K562/FLM cells upon ivermectin treatment can be an effective strategy for eradicating resistant populations.^35^, ^36^

Although our initial hypothesis suggested that the combined treatment of flumatinib and ivermectin could partially overcome flumatinib resistance in K562/FLM cells, repeated experiments consistently demonstrated no enhanced cytotoxic effects with this combination. Our findings indicate that ivermectin alone is capable of overcoming flumatinib resistance, making it a promising single‐agent therapeutic option. The lack of synergistic effects observed with the combination treatment suggests that the mechanisms underlying flumatinib resistance and ivermectin‐mediated cytotoxicity may not be directly linked. These results highlight the potential of ivermectin as an independent therapeutic strategy to optimize the treatment outcomes of CML patients with flumatinib resistance. Further investigations are warranted to elucidate the precise molecular mechanisms underlying the efficacy of ivermectin in overcoming resistance and to explore its potential clinical applications in CML treatment.

In conclusion, the study's extensive findings reveal complex, multifaceted mechanisms of chemotherapy resistance in K562/FLM cells, including efflux transporters overexpression, increased autophagy, alterations in signalling pathways and adaptative responses to high flumatinib concentrations. These insights emphasize the need for combination treatments that target not only the primary mode of action of TKIs but also secondary resistance mechanisms. Ivermectin emerges as a potential therapeutic agent that could be used to overcome resistance in leukaemic cells. Further studies are needed to unravel the underlying molecular mechanisms of ivermectin's action in these resistant cells.

## AUTHOR CONTRIBUTIONS

**Jixian Huang:** Conceptualization (equal); funding acquisition (lead); investigation (equal); methodology (equal); writing – original draft (equal). **Jie Xiao:** Conceptualization (equal); data curation (equal); methodology (equal). **Lifeng He:** Conceptualization (equal); data curation (equal). **Wenjie Dai:** Conceptualization (equal); formal analysis (equal); methodology (equal). **Jian Xiao:** Conceptualization (equal); formal analysis (equal). **Yuquan Li:** Conceptualization (equal); formal analysis (equal). **Ying He:** Conceptualization (equal). **Liang Yu:** Data curation (equal); funding acquisition (equal); investigation (lead); methodology (equal); writing – original draft (equal); writing – review and editing (lead).

## FUNDING INFORMATION

This work was supported by the Shaoguan City Science and Technology Plan Project (No. 210802154537494), Shaoguan City Health and Wellness Research Project (No. Y23061 and Y21042).

## CONFLICT OF INTEREST STATEMENT

The authors confirm that there are no conflicts of interest.

## Data Availability

The data that support the findings of this study are available from the corresponding author upon reasonable request.
